# Speech and language therapists’ perceptions of corporate practice in South Africa

**DOI:** 10.4102/sajcd.v72i1.1100

**Published:** 2025-06-13

**Authors:** Ntandoyenkosi L. Msomi, Suvishka Barath, Andrew J. Ross

**Affiliations:** 1 Department of Family Medicine, College of Health Sciences, University of KwaZulu-Natal, Durban, South Africa

**Keywords:** corporate practice, speech and language therapy, cultural competence, South Africa, cultural sensitivity

## Abstract

**Background:**

Corporate speech and language therapy (CSALT) is an emerging field that extends the role of speech and language therapists (SALTs) into corporate environments, enhancing workplace communication and interpersonal skills. While internationally recognised, CSALT remains underexplored in South Africa, where linguistic and cultural diversity present challenges and opportunities for its integration.

**Objectives:**

This study aimed to explore South African SALTs’ perceptions of CSALT.

**Method:**

A qualitative research approach was employed, grounded in a constructivist paradigm. Semi-structured interviews with seven SALTs were conducted and analysed using inductive thematic analysis. The study was conducted through remote interviews via Microsoft Teams.

**Results:**

Participants emphasised the relevance of CSALT in enhancing workplace communication, particularly in multilingual and culturally diverse corporate environments. Essential competencies for effective CSALT practice included business acumen, voice training and cultural competence, with a strong focus on understanding corporate communication styles. However, several barriers to CSALT were noted, such as limited public awareness, misconceptions about CSALT, accessibility challenges and insufficient academic preparation. Participants highlighted the need for greater recognition of CSALT and the development of more inclusive service delivery models to support its integration.

**Conclusion:**

CSALT can potentially improve workplace communication and inclusivity in South Africa. However, its growth is hindered by accessibility challenges and a lack of CSALT-specific training. Addressing these barriers could support its integration into national workforce development strategies.

**Contribution:**

This study highlights the potential to expand SALTs’ scope of practice and contribute to the global discourse on speech and language therapy in corporate health.

## Introduction

The provision of speech and language therapy services within corporate settings is gaining attention, particularly in the global North, as organisations increasingly recognise the importance of effective communication for enhancing employee productivity and achieving corporate objectives (Feinstein-Whittaker et al., 2012). Corporate speech and language therapy (CSALT) is an emerging practice area where speech and language therapists (SALTs) extend their services beyond traditional settings, such as public and private healthcare institutions, academic environments and educational institutions. In those settings, the primary focus has been on rehabilitating individuals presenting with developmental and acquired communication disorders (Feinstein-Whittaker et al., 2012). SALTs are uniquely trained healthcare professionals in communication, speech production, language and sociolinguistics (Antoni, 2017). The aforementioned areas make SALTs knowledgeable in various aspects of human communication. Their role can be similar to that of vocal coaches and anthropologists in aspects such as voice performance and sociocultural needs, respectively (Mason, 2018; Purser, 2021). The unique contribution of SALTs in corporate environments is the understanding of human communication across the life span and remedial and intervention strategies to attain social, cultural and communication aspects (Antoni, 2017).

The roots of CSALT can be traced back to the mid-1990s when a significant influx of non-native English speakers into the United States (US) created a pressing need for practical communication training in various industries, including call centres, hospitality and manufacturing (Schwartz, 2015). These non-native speakers faced unique communication challenges that affected the corporate culture, workplace performance and overall business practices. At that time, native English teachers were primarily viewed as critical resources for helping employees develop the requisite communication skills (Schwartz, 2015). While these educators possessed strong linguistic foundations and teaching experience, SALTs’ engagement in this realm remained unexplored.

Communication training has since evolved into a crucial element of employee development programmes. Many organisations now recognise that strong oral and written communication skills are fundamental for individual career success and achieving broader organisational goals (Bucăţa & Rizescu, 2017). Practical communication training typically encompasses conflict resolution, interpersonal communication, interviewing techniques, group discussions and oral presentations (Hargie, 2021). Employee work performance policies in the US indicate that such training significantly enhances workplace effectiveness, facilitating essential functions, such as planning, organisation, collaboration and leadership (Solomon & Theiss, 2022). For instance, a managerial skills training programme identified verbal communication skills as a significant factor in job rank, while written communication skills were linked to career advancement opportunities (Solomon & Theiss, 2022). Additionally, a survey of industry stakeholders highlighted that self-motivation, problem-solving abilities and strong oral communication skills were among the most valued competencies for entry-level positions (Ortiz et al., 2016). In South Africa, while specific employee work performance policies might not be as widely documented or standardised as in some international contexts, some corporate sectors and substantial multinational companies have begun integrating communication training into employee development programmes (Weideman & Hofmeyr, 2020). However, such initiatives are still emerging and may be inconsistent across industries (Govender & Bussin, 2020).

Despite the established importance of communication in corporate environments, the practice of CSALT remains underexplored, particularly in South Africa. The country’s diverse cultural and linguistic landscape presents unique challenges and opportunities for integrating CSALT into corporate health infrastructure and development policies. South Africa’s historical context, characterised by complex socio-linguistic dynamics and socio-economic disparities (Turner, 2020), further necessitates an understanding of how these factors influence the delivery and effectiveness of speech and language therapy services in corporate settings (Wigdorowitz et al., 2022). Given that CSALT is an emerging field in South Africa, understanding the perceptions of SALTs without prior exposure to it is crucial. These perceptions may influence the field’s acceptance, future uptake and integration into mainstream SALT practice. By exploring the perceptions of SALTs regarding CSALT, this study aimed to highlight the potential of CSALT to enhance communication, inclusivity and workplace productivity within the South African context.

## Research methods and design

### Study design

This study utilised a qualitative research approach grounded within a constructivist paradigm to obtain information reflecting participants’ perceptions using broad-based questions and allowing for the emergence of new information (Msomi & Ross, 2024; Pilarska, 2021).

### Participants

Convenience sampling was used to recruit study participants, and the potential participants were accessed with the assistance of Facebook and WhatsApp group administrators. Convenience sampling was used to select participants who were registered SALTs working in various traditional settings but with no work experience in CSALT. These participants were chosen to explore baseline perceptions of CSALT among professionals not yet exposed to the field and to identify factors that may influence its awareness, acceptance and potential future adoption.

The inclusion criteria were as follows:

- Registered with the Health Professions Council of South Africa as an SALT in the independent practitioner category.
- Practising in public healthcare institutions, educational institutions or private practices.

The exclusion criteria were as follows:

- Dually qualified audiologist and SALT, registered with the Health Professions Council of South Africa.

Seven SALTs consented to participate in the study (Table 1). All had obtained their B. Speech and language therapy qualification from the University of KwaZulu-Natal and had no previous work experience in CSALT.

**TABLE 1:** Characteristics of participants.

Participant code	Age (years)	Gender	Current work sector	Years of SALT experience
P1M25	25	Male	Private Healthcare	3
P2M24	24	Female	Education	2
P3F24	24	Female	Private Healthcare	3
P4F26	26	Female	Private Healthcare	4
P5F27	27	Female	Education	5
P6F23	23	Female	Education	2
P7M22	24	Male	Private Healthcare	2

Note: Participant code: P, participant; M, male; F, female.

SALT, speech and language therapist.

### Data collection

A recruitment poster was disseminated through designated closed Facebook and WhatsApp groups specific to SALTs. Participants who were interested and met the inclusion criteria directly texted the researcher. Thereafter, potential participants were emailed the study information and consent form. Interviews were conducted with the participants remotely via Microsoft Teams.

The qualitative data were collected using a semi-structured interview guide that facilitated open-ended discussions with minimal interviewer influence. This approach allowed participants to express their views freely and spontaneously while the researcher used prompts to explore emerging themes (Archard & O’Reilly, 2023). The first author provided probe questions to participants that yielded an expanded volume of qualitative data to increase sample sufficiency and to obtain salient themes. A widely recognised benchmark for qualitative sample size is achieving thematic saturation (DeJonckheere & Vaughn, 2019). However, Weller et al. (2018) suggested that focusing on item salience is a more practical approach for assessing the adequacy of sample size rather than relying solely on the point of thematic saturation.

The guide included the following questions:

1. What is your understanding of CSALT?
2. Do you think CSALT is necessary or relevant within the South African context? Please share your understanding with examples.
3. Did you learn about CSALT in university? Please elaborate on what you learned.
4. What competencies or skills do you think are needed to offer CSALT services in South Africa? Please share some examples.
5. How adequately prepared do you feel you can offer the following services to businesses on a scale of 1–10?
  5.1 Voice training.
  5.2 Social skills training.
  5.3 Respiratory training.
  5.4 Accent modification training.
6. How can CSALT be incorporated into the curriculum?
7. What challenges do you foresee when implementing CSALT services in South Africa?

### Data analysis

The data analysis process involved transcribing semi-structured interviews verbatim by the first author to ensure the accuracy and reliability of the transcriptions, wherein a second author independently reviewed a subset of the transcriptions. Any discrepancies or errors identified during this review were corrected before the transcriptions were imported into NVivo 12 software for coding. Inductive thematic analysis, following the six-step approach outlined by Braun and Clarke (2006), was employed to identify common themes and subthemes. The first author (N.L.M.) read the transcripts repeatedly to become familiar with the raw data and gain a deep understanding of the content.

Subsequently, a coding framework was developed and applied consistently across all transcripts. The analysis began with line-by-line coding, creating free codes that were later grouped into broader categories, resulting in the identification of themes and subthemes. This inductive approach was particularly suitable given the limited prior research on CSALT in South Africa, allowing for insights to emerge directly from the data.

To ensure consistency and accuracy, the first (N.L.M.) and second (S.B.) authors held regular meetings to discuss preliminary themes and subthemes, refining them by examining the nuances and discrepancies within the data. This collaborative approach ensured a comprehensive exploration of the data and allowed for the identification of deeper patterns that might have otherwise been overlooked.

The process of refining the themes involved ensuring that each theme had sufficient, relevant data to support it. The final thematic map provided a clear and internally consistent account of the perceptions of South African SALT regarding CSALT, offering valuable insights into its integration within corporate environments.

### Trustworthiness

The Lincoln and Guba’s Model of Trustworthiness was applied to this study (Enworo, 2023). Lincoln and Guba’s Model of Trustworthiness outlines four key criteria that researchers should focus on to ensure the quality and credibility of qualitative research findings, these being credibility, dependability, confirmablitty and transferability. These criteria help establish the study’s trustworthiness, mainly when dealing with subjective interpretations and perceptions (Enworo, 2023). The study ensured credibility through extensive engagement with the data, which included member checking to verify the accuracy and interpretation of findings. After the data analysis was completed and preliminary themes were identified, participants were invited to review a summary of the findings. This involved sending each participant a brief overview of the themes and subthemes from their interviews via a blind email. Participants were asked to confirm whether these themes accurately reflected their views and to provide any additional feedback or clarification. No significant disagreements were identified in the feedback. However, a few participants suggested minor wording refinements to portray better their perceptions, particularly regarding the public conception of CSALT. These suggestions were incorporated into the final thematic descriptions to ensure great authenticity. This feedback process strengthened the credibility of the findings and aligned with the participants’ intended meanings.

Dependability was addressed by providing detailed descriptions of the study methods, thereby ensuring consistency in the research process. Researchers achieved confirmability through independent data analysis, which utilised researcher triangulation to enhance the trustworthiness of the findings. Transferability was supported by comprehensive descriptions of the participants and the study context, allowing for the applicability of the findings to similar settings. Reflexivity was ensured in the study, whereby the first author (N.L.M.) was aware of his influence on the participants because of professional overlap, as he is also a practising SALT. The first author (N.L.M.) allowed independent expression among all participants, acknowledged their viewpoints and did not engage in counterarguments (Creswell, 2015).

### Ethical considerations

Full ethical approval was received from the Humanities and Social Sciences Ethics Committee on the 25 October 2024 from the University of KwaZulu-Natal (approval number: HSSREC/00007641/2024). The confidentiality of participants’ data was safeguarded through several measures, including password-protected computer files and consent forms secured with passwords. Personal identifiers were removed from all study materials, and codes were assigned to participants. Each participant was provided verbal and written explanations of the research, emphasising voluntary participation and their right to withdraw from the study at any time without penalty. Participation in the study posed no risks to SALTs. However, participants were required to provide signed consent for the interviews and for their information to be used anonymously in the research.

## Results

Three themes and nine subthemes derived from the analysis. Table 2 provides a summary of the themes and subthemes.

**TABLE 2:** Themes and subthemes.

Themes	Subthemes	Summary
1. Relevance of CSALT within the South African corporate health infrastructure	1. Conceptualisation of CSALT	CSALT is seen as expanding speech and language therapy into business settings, enhancing professional communication beyond clinical or educational roles.
	2. Addressing cultural and linguistic diversity	CSALT could bridge communication gaps in South Africa’s diverse corporate environments to address language and cultural differences among professionals.
	3. Promoting inclusivity and communication equity	CSALT is regarded as a means to promote fair and accessible communication across workplace language and cultural barriers.
2. Competencies required for CSALT practice	1. Business acumen and corporate communication skills	CSALT practitioners need to understand corporate language and etiquette to support clients in navigating professional communication demands.
	2. Advanced voice and articulation training	Expertise in voice modulation, articulation and tone control was essential for helping corporate clients communicate effectively.
	3. Cultural competence and interpersonal skills	Understanding non-verbal cues and cultural nuances is vital for CSALT practitioners working with diverse corporate clients.
	4. Gaps in academic preparation for CSALT	Little CSALT training, highlighting a need for more exposure to corporate applications in speech and language therapy education.
3. Challenges in CSALT implementation	1. Public perception of CSALT	SALT is generally viewed as a clinical field, which could hinder the acceptance of CSALT in business environments.
	2. Accessibility and cost barriers	CSALT may be perceived as an elite service accessible only to high-income professionals, impacting its broader adoption and accessibility.

CSALT, corporate speech and language therapy; SALT, speech and language therapy.

### Theme 1: Relevance of corporate speech and language therapy within the South African corporate health infrastructure

Participants emphasised CSALT’s importance in South Africa, mainly because of the country’s rich cultural and linguistic diversity. CSALT plays an important role in bridging workplace communication gaps and addressing challenges arising from cultural differences and varied language backgrounds. This theme had three subthemes, these being the conceptualisation of CSALT, cultural and linguistic diversity and inclusivity and communication equity.

#### Subtheme 1.1: Conceptualisation of corporate speech and language therapy

Participants described CSALT as expanding the scope of speech and language therapy into corporate settings. Unlike conventional speech and language therapy, which often targets speech and language disorders, CSALT emphasises developing communication skills relevant to business settings, such as presentation skills, articulation and professional demeanour. This shift reimagines the role of speech and language therapy in addressing corporate communication demands:

‘Corporate speech and language therapy is about being placed in the corporate world, like seeing doctors or psychologists … but now, supporting business professionals.’ (P1M25)

‘It is using speech and language therapy to help people in corporate better their communication, like for presentations or social interactions.’ (P3F24)

‘We are not just dealing with speech issues; it is helping people improve how they come across in meetings or presentations.’ (P6F23)

#### Subtheme 1.2: Addressing cultural and linguistic diversity

Given South Africa’s multilingual and multicultural context, participants viewed CSALT as essential for supporting professionals who are navigating diverse workplace environments. They noted that CSALT could assist individuals in communicating across language and cultural divides, making it especially relevant for companies with diverse client bases or multicultural teams:

‘South Africa’s diversity means corporate speech therapists could bridge cultural and linguistic gaps in business.’ (P1M25)

‘Imagine someone presenting a business idea in Limpopo… learning CSALT would help with understanding local language and culture.’ (P3F24)

‘CSALT can be particularly helpful here because our workforce is so mixed, culturally and linguistically.’ (P7M22)

#### Subtheme 1.3: Promoting inclusivity and communication equity

Participants recognised CSALT as promoting inclusivity by ensuring that all professionals can effectively participate in corporate environments, regardless of their language or cultural background. CSALT was seen as enhancing equity by providing individuals with the tools to overcome communication barriers, thus contributing to a more inclusive work environment:

‘With so many languages and accents here, CSALT could make workspaces more inclusive by helping people express themselves fully.’ (P5F27)

‘It is essential, especially for people who might feel “different” in how they communicate at work.’ (P2M24)

‘CSALT can support inclusivity by reducing misunderstandings that stem from language differences.’ (P6F23)

### Theme 2: Competencies required for corporate speech and language therapy practice

This theme highlights the specific skill set that participants believe is necessary for CSALT to have, which combines traditional speech and language therapy techniques with knowledge of corporate communication and cultural sensitivity. This theme consisted of four subthemes, these being business acumen and corporate communication skills and advanced voice and articulation training.

#### Subtheme 2.1: Business acumen and corporate communication skills

Participants emphasised the importance of understanding corporate language, professional culture and workplace etiquette to increase business efficiency. This knowledge allows CSALT practitioners to better support clients within corporate settings, where communication styles and expectations differ significantly from other contexts:

‘You would have to know corporate lingo and understand the business setting.’ (P1M25)

‘Business skills … like knowing how to talk to businesspeople or understanding their culture, are essential.’ (P3F24)

‘It is a different world, you need to know the language of the boardroom, not just therapy.’ (P6F23)

#### Subtheme 2.2: Advanced voice and articulation training

Participants highlighted voice and articulation skills as central to CSALT, with many corporate clients requiring support with voice modulation, clear articulation and the ability to convey authority, making these skills essential for CSALT practitioners:

‘In CSALT, knowing voice techniques such as intonation, tone, when to pause would be key.’ (P2M24)

‘Voice training and articulation are things I would focus on to help clients present themselves well.’ (P4F26)

‘People need help with not just what to say, but how they sound when they say it.’ (P7M22)

#### Subtheme 2.3: Cultural competence and interpersonal skills

Given the diverse nature of the South African workplaces, participants underscored the importance of cultural competence for CSALT practitioners. This involves sensitivity to cultural norms and understanding nonverbal communication cues, which vary across cultural backgrounds:

‘Being culturally aware is vital … we need to help people understand each other despite different backgrounds.’ (P1M25)

‘CSALT involves nonverbal skills and understanding cues that are not universal.’ (P5F24)

‘We have to consider different cultural approaches to communication, not just voice.’ (P6F23)

#### Subtheme 2.4: Gaps in academic preparation for corporate speech and language therapy

The current university curriculum primarily focuses on clinical applications of speech and language therapy, leaving participants feeling underprepared for CSALT roles in corporate environments. Participants reported that their university training focused almost exclusively on clinical and educational settings, with minimal exposure to corporate or non-traditional speech and language therapy applications. This section is about the implications of limited exposure; the next section is about what is needed:

‘There was nothing specifically on corporate speech and language therapy during my degree.’ (P2M24)

‘We did voice work but not specifically for corporate settings or for CSALT.’ (P4F26)

‘We were not trained for this; everything was clinical.’ (P7M22)

### Theme 3: Challenges in corporate speech and language therapy implementation

This theme explores the obstacles that participants anticipated in implementing CSALT, including public misconceptions of speech and language therapy as purely clinical and concerns over the accessibility and affordability of CSALT services. This theme consisted of two subthemes, these being the public’s perception of CSALT and accessibility and cost barriers.

#### Subtheme 3.1: Public perception of corporate speech and language therapy

Participants noted that the public often perceives speech andl anguage therapy as relevant only to clinical settings, which could limit CSALT’s acceptance within corporate environments. They emphasised the need for awareness efforts to showcase CSALT’s value in enhancing professional communication:

‘People think speech therapists only work with speech disorders so that CSALT may need more awareness.’ (P4F26)

‘It is hard to see CSALT accepted if people do not think speech-language therapy has a place in business.’ (P1M26)

‘There is a misconception that speech and language therapy is only for kids or people with speech issues.’ (P6F23)

#### Subtheme 3.2: Accessibility and cost barriers

Participants also raised concerns about the perception of CSALT as an elite service, suggesting that it may be accessible primarily to high-income individuals or large corporations. They advocated for efforts to make CSALT more inclusive and accessible, potentially through partnerships with businesses or sliding-scale fee structures:

‘CSALT could get seen as something exclusive or “extra,” which means not everyone can access it.’ (P5F27)

‘It is about accessibility, so we need ways to offer CSALT to everyone, not just executives.’ (P3F24)

‘Making it affordable is crucial; otherwise, it might only serve a privileged few.’ (P7M22)

## Discussion

According to the authors’ knowledge, this study is among the first to explore South African SALT’s perceptions of CSALT, offering insights into how this practice is conceptualised, the skills deemed necessary and the anticipated challenges in implementing it within a multicultural corporate landscape. Participants illustrate a vision for CSALT as an evolving area of speech and language therapy practice uniquely suited to South Africa’s corporate landscape.

Participants viewed CSALT as a significant expansion of traditional speech and language therapy, focusing on enhancing corporate communication rather than addressing clinical needs. This aligns with international trends, where CSALT is recognised for supporting corporate clients in developing professional communication skills, including voice modulation, articulation and presentation skills (Specht & Blanchet, 2009). However, unlike countries where CSALT is established, participants in this study highlighted that in South Africa the field is still emerging, with limited public awareness and academic training available. CSALT was described as moving beyond traditional clinical roles to serve professionals in the workplace, reflecting a shift in the perceived value of speech and language therapy. This aligns with global discussions on the versatility of speech and language therapy across diverse professional fields (Abrahams et al., 2023). For instance, CSALT’s emphasis on presentation, voice control and corporate social skills demonstrates the field’s adaptability and relevance in non-clinical spaces, providing exciting, flexible alternatives to traditional SALT positions (Specht & Blanchet, 2009). This expansion could fill a unique niche in South Africa, particularly as companies seek ways to enhance internal communication and interpersonal dynamics. The study highlighted that CSALT primarily benefits individuals in professional roles who do not necessarily have speech disorders but seek improved communication in work settings. This supports previous research suggesting that CSALT can enhance confidence and professionalism among corporate clients (Specht & Blanchet, 2009). However, the South African context presents unique challenges, as these services must cater to a linguistically and culturally diverse population (Mophosho, 2018). Tailoring CSALT to meet these varied needs could expand its impact and relevance in South African workplaces.

An emphasis on CSALT’s potential to bridge communication gaps within South Africa’s culturally diverse corporate environment was shared. This is particularly pertinent in South Africa, where workplaces often consist of multilingual and multicultural teams and communication misunderstandings can arise because of differences in language and cultural norms (Mophosho, 2018). CSALT’s ability to address these issues underscores its value in promoting inclusivity and enhancing interpersonal relations within professional settings. Participants viewed CSALT as an effective way to mitigate language barriers and cultural misunderstandings that may arise in corporate environments. This finding supports a study in sub-Saharan Africa, highlighting the importance of culturally sensitive communication training in diverse work settings to adapt to a genuinely diverse multilingual and multicultural context (Lüdtke, 2023).

In South Africa, CSALT practitioners could play a role in facilitating understanding across cultural lines, thus enhancing collaboration and reducing potential conflicts in diverse teams. Incorporating cultural sensitivity training into CSALT practices could further strengthen its impact. The wellness management policy for public service in South Africa requires suitably qualified healthcare professionals to render therapeutic services to employees on all duty levels, with a focus on enhancing work productivity (Mkansi, 2019). CSALT can support inclusivity by ensuring that all employees, regardless of language or cultural background, have equal communication opportunities. This aligns with global calls for workplace inclusivity and equity, highlighting the need for communication skills that accommodate diverse voices and perspectives (Chukwudi & Eusebius, 2023). CSALT in South Africa could uniquely position SALTs as advocates for equitable communication, fostering workplaces that value diverse contributions and reducing biases in communication. This study identified specific skills deemed essential for CSALT, blending traditional speech and language therapy expertise with corporate communication and cultural competence. These findings highlight the need for CSALT practitioners who can navigate the demands of corporate environments and support clients in refining verbal and nonverbal communication.

The importance of understanding corporate jargon, workplace etiquette and professional norms indicates that CSALT practitioners require a strong foundation in corporate communication. This contrasts with traditional speech and language therapy, which focuses more on clinical or educational settings. Corporate speech and language therapy practitioners in South Africa may need additional training to understand the nuances of business interactions, making business acumen a critical area for curriculum development in South African speech and language therapy. Voice control and articulation are central to CSALT, specifically focusing on helping clients improve their public speaking and presence. In South Africa’s multilingual context, practitioners may need to tailor voice and articulation techniques to respect linguistic diversity, adapting approaches that suit the distinct phonetic and intonational patterns of various languages spoken in South African workplaces (Mason, 2018).

Cultural competence was seen as essential, particularly for CSALT in South Africa, where practitioners must understand diverse cultural communication styles. South African CSALT practitioners would benefit from training that includes nonverbal communication cues, cultural sensitivity and skills for adapting communication approaches to align with cultural norms (Schwartz, 2015). This theme highlights participants’ reflections on their academic training, which focused primarily on clinical and educational settings with minimal preparation for corporate roles. The limited CSALT exposure in academic programmes underscores the need for curriculum enhancements that align with the growing demand for CSALT in South Africa. An overall feeling of under-preparedness for CSALT because of the lack of targeted coursework in corporate applications. Studies from countries with established CSALT practices indicate that academic programmes increasingly include modules on business communication and voice training (Solomon & Theiss, 2022). Adapting similar coursework in South African programmes could better equip graduates for diverse professional roles, addressing a fundamental gap identified by participants. There is a need for CSALT-focused electives or modules, which could provide a foundation in corporate communication and business etiquette. By integrating CSALT into South African academic programmes, training institutions could produce graduates who are well versed in corporate applications of speech and language therapy, thus advancing CSALT’s development in the region.

While participants expressed optimism about CSALT’s potential, they highlighted several challenges, including public misconceptions about speech and language therapy only being a clinical field, and concerns over accessibility and affordability. Addressing these challenges will be essential for CSALT to gain traction within South African corporate environments (Bucăţa & Rizescu, 2017). Participants noted that the public often perceives speech and language therapy as limited to clinical or educational contexts, potentially hindering CSALT’s acceptance in corporate spaces. This aligns with global studies indicating that public awareness is a barrier to expanding speech and language therapy into non-traditional settings (Schwartz, 2015). Public awareness campaigns could help reshape perceptions of CSALT in South Africa, demonstrating its relevance for professional communication and positioning it as a valuable corporate service. Participants expressed concerns about CSALT being viewed as an elite service accessible only to high-income clients, potentially excluding those in lower-income or entry-level roles. South African CSALT practitioners may need to explore partnerships with corporations or offer scalable pricing models to overcome this barrier. Making CSALT affordable could broaden its impact and foster a more inclusive approach, in line with calls for equitable access to professional development services.

### Implications and recommendations

This study revealed key insights with significant implications for corporate health infrastructure development. The corporate sector should acknowledge the value of CSALT and actively integrate these services into their employee wellness programmes. CSALT can play a pivotal role in improving communication skills, fostering team collaboration, enhancing conflict resolution and promoting overall employee well-being, which in turn can drive organisational success. To ensure effective implementation, partnerships between higher education institutions and the corporate sector must be strengthened, particularly in the area of student training for CSALT. Collaboration through initiatives such as student placements within employee wellness clinics and telehealth services, enabling students to gain hands-on experience while helping to meet the needs of corporate clients, is recommended.

Future research should consider exploring the perspectives of corporate clients who have benefitted from CSALT services to provide a more comprehensive understanding of its impact on workplace communication and productivity. Future studies could also explore the long-term effects of CSALT on employee performance and mental well-being or investigate sector-specific challenges and opportunities for CSALT implementation.

### Limitations

SALTs were recruited from closed Facebook and WhatsApp groups, which limited the sample frame. This influenced the diversity of perceptions captured in the study. While the study specifically aimed to explore the perceptions of SALTs regarding CSALT, this also means that the findings reflect only their assumptions and theoretical understanding rather than reflections from actual CSALT practice. Perceptions may differ significantly once SALTs gain practical exposure to CSALT in the workplace. The study primarily focused on SALTs in South Africa, and therefore, the findings may not apply to other countries with different linguistic contexts and socio-cultural and economic factors. The integration of CSALT into corporate environments could vary widely in different countries.

## Conclusion

This study reveals the untapped potential of CSALT in South Africa and emphasises the need for targeted training, public awareness and inclusive service models to realise this potential. By addressing these challenges and leveraging CSALT’s ability to enhance communication in diverse corporate environments, South Africa can expand speech and language therapy’s impact beyond clinical settings, promoting inclusivity and professional development in the corporate sector.
